# Hematopoiesis: A Layered Organization Across Chordate Species

**DOI:** 10.3389/fcell.2020.606642

**Published:** 2020-12-16

**Authors:** Ramy Elsaid, Francisca Soares-da-Silva, Marcia Peixoto, Dali Amiri, Nathan Mackowski, Pablo Pereira, Antonio Bandeira, Ana Cumano

**Affiliations:** ^1^Unit of Lymphocytes and Immunity, Immunology Department, Institut Pasteur, Paris, France; ^2^INSERM U1223, Paris, France; ^3^Université de Paris, Céllule Pasteur, Paris, France; ^4^I3S—Instituto de Investigação e Inovação em Saúde and INEB—Instituto Nacional de Engenharia Biomédica, Universidade do Porto, Porto, Portugal; ^5^Instituto de Ciências Biomédicas Abel Salazar, Universidade do Porto, Porto, Portugal; ^6^Graduate Program in Areas of Basic and Applied Biology, Instituto de Ciências Biomeìdicas Abel Salazar, Universidade do Porto, Porto, Portugal

**Keywords:** hematopoieisis, lymphopoieis, embryo, evo devo biology, layered

## Abstract

The identification of distinct waves of progenitors during development, each corresponding to a specific time, space, and function, provided the basis for the concept of a “layered” organization in development. The concept of a layered hematopoiesis was established by classical embryology studies in birds and amphibians. Recent progress in generating reliable lineage tracing models together with transcriptional and proteomic analyses in single cells revealed that, also in mammals, the hematopoietic system evolves in successive waves of progenitors with distinct properties and fate. During embryogenesis, sequential waves of hematopoietic progenitors emerge at different anatomic sites, generating specific cell types with distinct functions and tissue homing capacities. The first progenitors originate in the yolk sac before the emergence of hematopoietic stem cells, some giving rise to progenies that persist throughout life. Hematopoietic stem cell-derived cells that protect organisms against environmental pathogens follow the same sequential strategy, with subsets of lymphoid cells being only produced during embryonic development. Growing evidence indicates that fetal immune cells contribute to the proper development of the organs they seed and later ensure life-long tissue homeostasis and immune protection. They include macrophages, mast cells, some γδ T cells, B-1 B cells, and innate lymphoid cells, which have “non-redundant” functions, and early perturbations in their development or function affect immunity in the adult. These observations challenged the view that all hematopoietic cells found in the adult result from constant and monotonous production from bone marrow-resident hematopoietic stem cells. In this review, we evaluate evidence for a layered hematopoietic system across species. We discuss mechanisms and selective pressures leading to the temporal generation of different cell types. We elaborate on the consequences of disturbing fetal immune cells on tissue homeostasis and immune development later in life.

## Layered Hematopoiesis: A Historical Perspective

Hematopoiesis, the process by which blood cells are produced, has been considered to be initiated by hematopoietic stem cells (HSC) that develop through multiple differentiation intermediates and give rise to all blood lineages. Recent studies have challenged this view by showing that specialized embryonic-derived subsets persist throughout life.

During embryogenesis, successive waves of mesoderm-derived hematopoietic progenitors contribute to the formation of erythroid, myeloid, and lymphoid lineages. In the 1920s, studies on embryonic hematopoiesis revealed that the first blood cells appear in the yolk sac (YS): red blood cells were found in clusters surrounded by an endothelial layer, named blood islands. The order of events that resulted in the formation of these structures led embryologists to hypothesize that both hematopoietic and endothelial cells had a common origin, a bipotent cell designated hemangioblast (see Table 1; Choi et al., 1998). In the 1970s, Moore and Owen (1967) showed that the embryonic day (E) 7 chicken YS generated mostly erythroid and also myeloid and lymphoid progeny after transplantation into irradiated embryos, pointing to a YS origin of HSCs. In mammals, the first single-cell assays showed the existence of multilineage progenitors in the YS, shortly after the beginning of circulation (Moore and Metcalf, 1970). Subsequent studies using chick–quail or chick–chick chimeras challenged the view that YS was the source of HSCs and showed that long-lasting hematopoietic potential was only found in intra-embryonic progenitors (Dieterlen-Lievre, 1975; Lassila et al., 1978). In mouse, culture of YS vs. the intra-embryonic aorta-gonad-mesonephros (AGM) region before the establishment of circulation demonstrated that the origin of HSCs is exclusively intra-embryonic (Cumano et al., 1996). It is now accepted that three waves of hematopoietic progenitors are generated in the mammalian embryo. The first wave is generated during gastrulation in the YS blood islands (Godin and Cumano, 2002). Soon after, a second wave of progenitors emerges in the YS vascular plexus (Frame et al., 2016). These two hematopoietic waves, which generate primitive and definitive erythrocytes, respectively, provide the oxygen needed for embryo survival as well as megakaryocytes and myeloid cells that are important for tissue remodeling and hemostasis. The third wave occurs in the great vessels of the embryo and generates HSCs that will migrate to the fetal liver (FL) where they expand (Ema and Nakauchi, 2000). This pool of HSCs will sustain hematopoiesis throughout adult life, generating not only erythroid and myeloid cells but also lymphoid cells.

**TABLE 1:** Glossary.

**Hemangioblast**. A bipotent hematopoietic and endothelial progenitor. The most common example is yolk sac blood islands in mice where primitive erythrocytes and the endothelial cells lining these structures are thought to have a common progenitor. In the mouse embryo, multiple progenitors contribute to blood island formation, and therefore, their common origin remains to be formally demonstrated. **Endothelial-to-hematopoietic transition (EHT)**. The process whereby cells with morphology, phenotype, and spatial position of endothelial cells convert into a hematopoietic cell. This process has been identified to be at the origin of erythromyeloid bipotent cells in the vasculature of the yolk sac and of multipotent hematopoietic stem cell progenitors in the dorsal aorta. This conversion has been clearly visualized in the zebrafish embryo and is independent of cell division. In the fish embryo, newly converted single hematopoietic cells appear to rapidly egress into the space between the vein and the artery. By contrast, in mammalian and chicken embryos, newly converted cells extensively expand *in situ* forming hematopoietic intra-aortic clusters budding into the lumen, before being released into blood circulation. **Hemogenic endothelium**. Designates the endothelial cells in the yolk sac and dorsal aorta that have the capacity to convert into a hematopoietic cell through a process named EHT. **Hematopoietic intra-aortic clusters (HIAC)**. Clusters of hematopoietic cells adjacent to the endothelium protruding in the lumen of the dorsal aorta after being generated through EHT. They are found in birds and mammalian embryos, but not in zebrafish. **Primitive wave**. The first hematopoietic cells with a given identity. Designates the first hematopoietic cells that generate primitive erythrocytes. Primitive erythrocytes derive from erythroid progenitors in the YS blood islands and are large nucleated cells that express embryonic hemoglobins Hbb-bh1 and Hbb-y. This concept also includes macrophages and megakaryocytes. **Definitive wave**. Refers to the hematopoietic cells that generate definitive erythrocytes. These are enucleated cells that express embryonic and adult forms of hemoglobin (Hbb-bh1 and Hbb-b1) but lack Hbb-y hemoglobin expression. They can originate from either erythromyeloid progenitors that derive from the YS or from hematopoietic stem cells of intra-embryonic origin, during embryonic or post-natal life, respectively. **Somatic recombination**. Genomic recombination that occurs in somatic cells. It is the process whereby the genes coding for the antigen receptors in T and B lymphocytes are assembled from the random assortment of variable (V), diversity (D), and joining (J) elements on immunoglobulin heavy chain, TCRβ and TCRδ chains, or from V and J elements in Ig light chains, TCRα and TCRγ chains. **T cell negative selection**. Elimination of T lymphocytes expressing a self-reactive antigen receptor occurring in the thymus. Negative selection is mediated through the interaction of immature thymocytes with thymic epithelial cells located in the thymic medulla (mTEC) and dendritic cells (DC) that express a large collection of tissue-specific peptides. **Antigen receptor repertoire**. Collection of the diverse antigen receptors expressed by B or T lymphocytes and generated by somatic recombination (see above). The diversity of the antigen receptor repertoire is further increased by trimming of the ends of the segments and addition of nucleotides without templated (N sequence addition) prior to the joining. **Tolerance**. The absence of reactivity toward a given antigenic determinant. Often referred to self-tolerance, it designates the absence of lymphocytes that recognize antigen determinants from the organism where they reside. Defective tolerance induction results in autoimmune disorders.

The notion that YS populations are only produced and necessary during embryonic development has recently been challenged. Inducible lineage-tracing mouse models formally demonstrated that YS progenitors generate unique tissue-resident macrophage populations that persist throughout adulthood (Ginhoux et al., 2010; Gomez Perdiguero et al., 2015). These studies challenged the dogma that HSCs were the source of all hematopoietic lineages in the adult and provided evidence for the concept of a “layered” hematopoietic system.

Embryonic hematopoiesis is thus characterized by the partial overlap of distinct waves of progenitors that transit through different organs in embryonic and adult life. Each wave is well-orchestrated in time and space, each serving specific internal and external environmental needs. We will speculate on the selective advantages of a layered system across evolution that relates to hematopoiesis in general and to lymphopoiesis.

## Embryonic Hematopoiesis in Different Species: A Common Strategy to Make Blood

### Foreword

Hemocytoblasts or primordial HSCs, derived from mesoderm, generate multiple hematopoietic cell types and are found across species starting in coelomic invertebrates. Key transcription factors that modulate the divergence of different hematopoietic cell types are conserved. For example, orthologs of *Gata*, *Fog*, and *Runx* have been identified in *Drosophila*. These transcription factors regulate the production of plasmatocytes, which exert phagocytic activity, granulocytes, with metabolic activity and immune function, and oxygen-transporting red blood cells (Evans et al., 2003; Hartenstein, 2006). In contrast, Metazoa without coelom are devoid of well-defined mesoderm and have a gelatinous matrix between ectoderm and endoderm enclosing large numbers of amoebocytes. These cells move using pseudopodia and fulfill functions that differ among species, ranging from defending the organism against pathogens, digesting food, or disposing of waste.

A major accomplishment in the transition of invertebrate to vertebrate metazoans was the development of a closed circulatory system and that of a centralized pumping organ, the heart, which ensures a rapid distribution of oxygen and nutrients to tissues, and the deployment of immune surveillance in the organism. Most invertebrates rely on myoepithelial cells with contractile capabilities to ensure that cells transit from the hemal spaces (fluid-filled sinus without a lining) and channels to the tissues, collectively designated as an open circulatory system (Muñoz-Chápuli et al., 2005; Hartenstein, 2006). However, there are several exceptions to this rule. For example, annelids have a closed circulatory system with pumping muscular blood vessels. Molluscs, instead, have an open circulatory system and one (or several) centralized hearts composed of cavities (atria and ventricles).

The emergence of a closed circulatory system with a centralized heart is linked to the appearance of vascular endothelial cells, the origin of which has long been a matter of debate. It was hypothesized that endothelial cells originate from the amoebocytes that, in acoelomatic invertebrates, adhere to the basement membrane that lines the hemal cavities (Muñoz-Chápuli et al., 2005). This hypothesis links the development of endothelial and hematopoietic cells throughout vertebrate evolution. We analyzed here different model systems of chordates in which independent generations of hematopoietic cells and different primary hematopoietic organs have been documented (Table 2).

**TABLE 2:** Models to study hematopoiesis.

**Species**	**Advantages**	**Disadvantages**	**Methodologies used for the study of hematopoiesis**
Mouse	Broad availability of transgenic and gene-deficient strains	Species-specific differences in basic biology (e.g., replicative rate, DNA damage response, etc.) Inbred mouse strains do not account with genetic diversity; the choice of a specific genetic background can influence the observed phenotype	*In vitro* clonal assays (CAFCs, LTC-IC, CFU assays) Flow cytometry phenotyping Functional repopulation assays (competitive and non-competitive transplantation assays) Lineage tracing models Clonal analysis of lineage fate in native hematopoiesis (Sun et al., 2014) Single-cell transcriptomics and proteomic analysis
Human	Extensively characterized hematopoietic system Higher translational value for clinical applications	Limited sources of human hematopoietic cells and tissues Limited accessibility to steady-state human hematopoiesis: limited studies of human hematopoietic cells on their natural microenvironment; no clonal tracking possible out of transplantation setting *In vivo* xenotransplantation murine models only capture part of the cell-intrinsic properties of human hematopoiesis Cell-extrinsic aspects of human hematopoiesis are difficult to access and study *In vivo* assays are time-consuming	Characterization of hematopoietic populations by surface markers expression—flow cytometry (Notta et al., 2011, 2016) Evaluation of differentiation potential—*in vitro* colony-forming assays (Notta et al., 2016) *In vivo* functional repopulation assays in immunodeficient mice—xenograft models (Kamel-Reid et al., 1989; Beer and Eaves, 2015) Repopulation dynamics of HSCs in humans—post-transplantation clonal tracking (Scala and Aiuti, 2019) Single-cell transcriptomics and proteomic analysis
Zebrafish	Rapid and external development Embryo optical transparency Easy high-resolution optical imaging in live animals Large-scale genetic and chemical screens Several transgenic lines available (reviewed in Stachura and Traver, 2016)	Lack of antibodies for phenotypic characterization Lack of knock-in technologies Need to establish breeding standards; Inbreed and outbreed depression	Genome targeting (ZFNs, TALENs, CRISPR, and morpholino-mediated gene knockdown) to produce mutants of interest (reviewed in Sertori et al., 2016) Major blood lineages isolation by size and granularity using FACS (Traver et al., 2003) Hematopoietic cell transplantation (Traver et al., 2003, 2004; Hess et al., 2013) Stromal culture assays (Stachura et al., 2011; Wolf et al., 2017) Clonal methylcellulose assays (Stachura et al., 2011) Parabiotic embryos for cell migration and homing studies (Demy et al., 2013) High-resolution time-lapse live imaging (e.g., Bertrand et al., 2010; Kissa and Herbomel, 2010) Xenotransplantation (Hess and Boehm, 2016; Parada-Kusz et al., 2018) *In vivo* lineage tracing (e.g., Murayama et al., 2006; Jin et al., 2007; He et al., 2020)
Axolotl	Neoteny (no metamorphosis) Regeneration without scar tissue formation	Lack of antibodies for phenotypic characterization Gene manipulation difficult to perform Long periods of generation	Transplantation (Lopez et al., 2014)
Xenopus	Large embryo size Lineage tracing strategies Available chimeric procedures to determine cell origin	Lack of antibodies for phenotypic characterization Gene manipulation difficult to perform	Chimeras (Du Pasquier et al., 1989) Lineage tracing of blastomeres (Ciau-Uitz et al., 2000)
Chicken	Large egg size Amenable to surgical manipulation Quail–chicken chimeric system	Lack of antibodies for phenotypic characterization Lack of growth factors for *in vitro* cultures Gene manipulation technologies difficult to perform	Quail–chicken and chicken–chicken chimeras (Le Douarin, 1969) Corio-allantoid transplantation (Yvernogeau and Robin, 2017) Lineage tracing (Jaffredo et al., 2000)

*DIC, differential interference contrast; FACS, fluorescence-activated cell sorting; TALENs, transcription activator-like effector nucleases; ZFNs, zinc finger nucleases.*

### Cephalochordates (Amphioxus)

**FIGURE 1:**
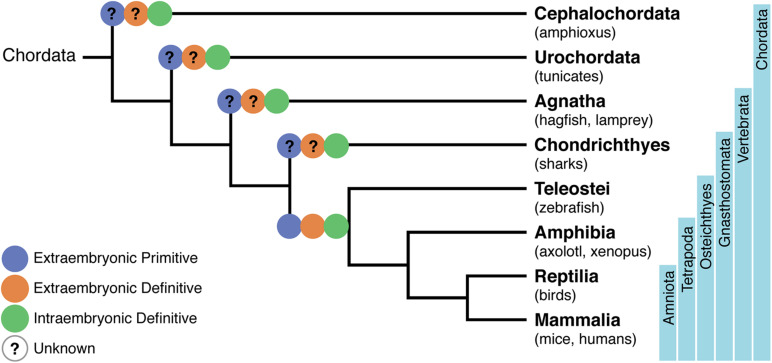
Phylogenetic tree of chordates showing the evolutionary relationships of the hematopoietic system. A phylogeny representing Cephalochordata to Amniota, illustrating the development of a layered hematopoietic system with (i) yolk sac (YS) or extra-embryonic primitive hematopoietic cell generation in blue, (ii) YS definitive hematopoietic cell generation in orange, and (iii) intra-embryonic definitive hematopoiesis in green. Osteichthyes (which include zebrafish, axolotl, Xenopus, birds, mice, and humans) were the first where the distinct origins of hematopoietic cells have been demonstrated. Lack of information on specific processes are represented with a question mark.

The amphioxus are invertebrates that together with Tunicates stand in close phylogenetic proximity to vertebrates (Delsuc et al., 2006; Figure 1). They appear, therefore, to be the model of choice to understand the evolution of a circulatory system. Developmental studies in amphioxus demonstrated that their cardiac region is decentralized and that blood is pumped by contractile vessels throughout life. By contrast, analysis of orthologs of *Pax2/5/8*, together with those of key vessel and hematopoietic development markers *Flk1* and *Scl*, identified specific expression in an AGM-like region, thus pointing to a similar origin of hematopoietic cells in amphioxus and vertebrates (Pascual-Anaya et al., 2013). This hypothesis was reinforced by experiments showing that the treatment of amphioxus larva with retinoic acid (RA) inhibited the expression of hematopoietic genes, reminiscent of what was observed in zebrafish larva and mouse embryonic stem (ES) cells where treatment with RA inhibited primitive hematopoiesis (Pascual-Anaya et al., 2013). Altogether, these data indicate that despite lacking a centralized heart, amphioxus developed a process that resembles hemogenic endothelium (HE) and endothelial-to-hematopoietic transition (EHT) (see Table 1). These studies were performed up to the stage of 2 day-old larva and, therefore, do not allow to assert whether invertebrates also generate hematopoietic progenitors at independent sites.

### Tunicates

Tunicates are currently considered as the closest relatives to vertebrates (Delsuc et al., 2006; Figure 1). Unlike cephalochordates, they have a centralized pumping organ (the heart) and an established network of blood vessels (Rosental et al., 2020). They have, therefore, in the sessile adult animal, circulating blood cells composed of myeloid cells. They also have a progenitor compartment located in the endostyle, tentatively considered as a functional equivalent to the bone marrow (BM) (Rosental et al., 2018). Cells endowed with cytotoxic activity that reject allografts could represent the equivalent population to NK cells. There is presently no experimental evidence on the origin of these cells.

### Agnathes or Jawless Chordates (Lampreys and Hagfish)

Lampreys possess circulating erythrocytes, myeloid and lymphoid cells, and were the most ancient vertebrates to show a robust adaptive immunity with the capacity to reject allografts, to produce specific antibodies, and to develop immunological memory. The molecular basis of this sophisticated immune system is a unique set of “building blocks” that encode lymphocyte antigen receptors (Pancer et al., 2004). They differ from those in higher vertebrates but are assembled in a similar manner, undergoing a process of somatic recombination (see Table 1) and exerting similar functions, illustrating an unprecedented example of parallel evolution. An independent site for T cell development, equivalent in function to the mammalian thymus, has been identified in the gills (Bajoghli et al., 2011). Lampreys have three different loci of the “building blocks” that encode antigen receptors of three distinct lymphoid cells reminiscent of the mammalian B, αβ-T, and γδ-T cells, indicating strong selective pressures for non-redundant functions of these different cell types (Boehm et al., 2018).

Lampreys develop through a complex life cycle with a larval stage, called ammocoetes, a metamorphosis that lasts for several months and an adult stage. In ammocoetes, the first hematopoietic cells are found in the typhlosole, which is a longitudinal fold of the intestinal inner wall, and also in the adipose tissue of the nephric fold. During metamorphosis, blood cell formation is displaced from these sites to the supra-neural body, a unique organ equivalent to the mammalian BM. The typhlosole is composed of mesenchymal cells located close to the dorsal aorta, and that form blood islands where the first hematopoietic cells are found. These hematopoietic cells appear by morphology to belong to the myeloid, erythroid, and lymphoid lineages and many of them are actively proliferating (Amemiya et al., 2007). Although it has proven difficult to identify, in lampreys, equivalent anatomical sites to the higher vertebrate YS or dorsal aorta (DA), it is possible that the first hematopoietic cells are generated in the typhlosole blood islands.

### Chondrichthyes or Cartilaginous Fish (Sharks)

Sharks are the most primitive organisms to have a functional adaptive immune system that is, unlike Agnathes, similar to that found in mammals. It is also in cartilaginous fish where the spleen is found for the first time in phylogeny and where hematopoietic progenitors were first detected and identified (Manca et al., 2018). It appears, therefore, that the spleen is the major hematopoietic organ already active in the embryo. The limited information concerning the hematopoietic development in these organisms suggests that HSC originated in the YS and transit later to the Leydig organ (located close to the esophagus) and to the spleen (Manca et al., 2019). In summary, there are different sites where hematopoiesis occurs over time, but the emergence of hematopoietic cells at multiple sites have not been documented.

### Teleosts or Bony Fish (Zebrafish)

Zebrafish (*Danio rerio*) is the most well-studied teleost species and has been an important model to study hematopoietic development, given that it is uniquely suitable for large-scale mutagenesis experiments, genome editing, chemical screenings, and high-resolution live imaging (Table 2).

**FIGURE 2:**
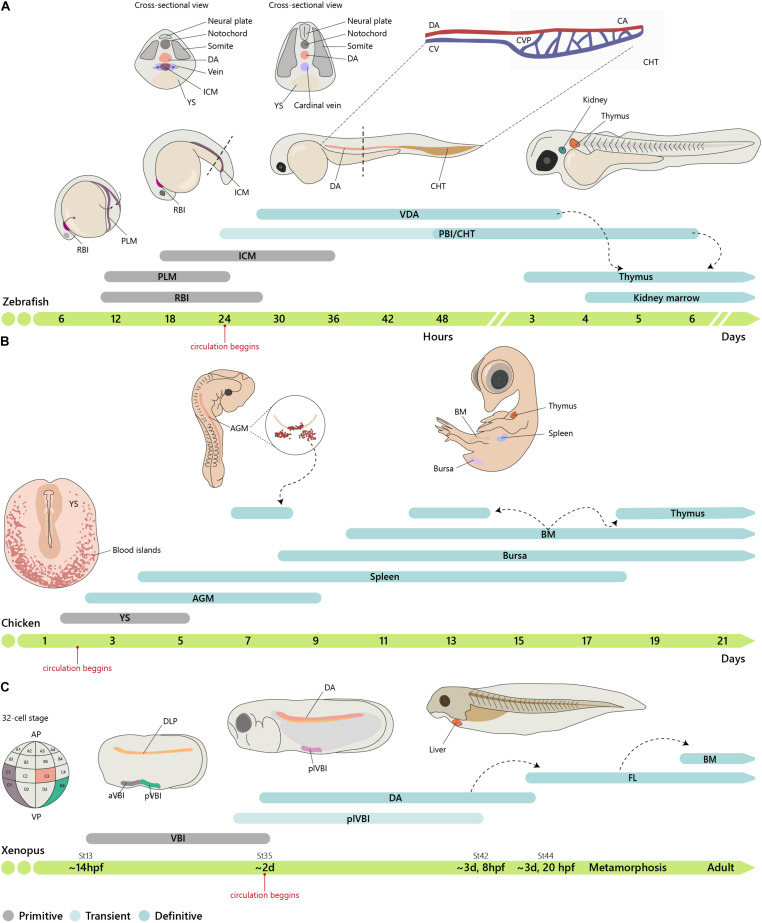
Embryonic origin of the hematopoietic system. **(A)** Timeline of hematopoietic development in zebrafish. In zebrafish, primitive hematopoiesis occurs in the RBI and ICM region generating primitive macrophages and erythrocytes, respectively. The CHT, consisting of the CA (the continuation of the DA as it enters the tail), CV, and an endothelial network in between, the CVP, hosts a niche for HSC expansion and differentiation, reaching a peak at around E6. **(B)** In chicken, primitive hematopoiesis *at* E1.5 occurs in the YS blood islands. IAHCs are first detected at E2.25, reach a peak at E3, and gradually decrease, being residual at E5.5. PAF cells are detected at E2.5, rapidly surpassing the number of HIAC and last until around E9. **(C)** In Xenopus, the first hematopoietic site is the VBI (YS equivalent). Subsequent generation occurs after progenitor cells from the DLP migrate to the midline where they coalesce to give rise to the dorsal aorta (AGM). Cells from the two waves colonize the liver, which is the definitive site of hematopoiesis in both larval and adult stages. Xenopus were staged according to Nieuwkoop and Faber. See http://www.xenbase.org/anatomy/alldev.do for equivalences to dpf. AGM, aorta-gonad-mesonephros; aVBI, anterior VBI; BM, bone marrow; CA, caudal artery; CHT, caudal hematopoietic tissue; CVP, caudal vein plexus; DA, dorsal aorta; ICM, intermediate cell mass; FL, fetal liver; *PAF, para-aortic foci;* PBI, posterior blood island; PLM, posterior-lateral mesoderm; pVBI, posterior VBI; plVBI, posterior-lateral VBI; RBI, rostral blood islands; YS, yolk sac; VBI, ventral blood island.

Similar to other vertebrates, zebrafish hematopoiesis develops through successive waves emerging at different locations (Table 3 and Figure 2). Contrary to mammals, birds, and other teleosts, zebrafish primitive hematopoiesis initiates intra-embryonically, in a structure known as intermediate cell mass (ICM). This structure results from the migration of two posterior bilateral stripes of lateral mesoderm (posterior-lateral mesoderm, PLM) to the trunk midline. It is in this structure where primitive erythroid progenitors and endothelial cells were found (Detrich et al., 1995; Thompson et al., 1998). Hematopoietic commitment, defined by the expression of the erythroid-specific transcription factor *Gata1*, occurs as early as the 2-somite stage [around 11 h post-fertilization (hpf)] in the PLM. Another site of primitive hematopoiesis was identified in the rostral blood island (RBI) region of the anterior mesoderm. This site produces macrophage-like cells that migrate into the yolk syncytial layer and disseminate through different tissues (Herbomel et al., 1999).

**TABLE 3:** Hematopoietic waves.

**Species**	**Adult hematopoietic sites**	**Embryonic hematopoietic waves**	**Location**	**Potential**
Mouse	Bone marrow Spleen	Primitive	YS	Primitive erythrocytes, macrophage colony-forming cells, and megakaryocyte colony-forming cells (Palis et al., 1999)
		Definitive	YS	Erythromyeloid progenitors (Palis et al., 1999; Bertrand et al., 2005)
			AGM	HSC (Cumano et al., 1996; Medvinsky and Dzierzak, 1996)
Human	Bone marrow	Primitive	YS	Primitive erythrocytes and myeloid cells (Tavian et al., 1999)
		Definitive	YS	Erythromyeloid progenitors (Migliaccio et al., 1986)
			AGM	HSCs (Tavian et al., 1996)
Zebrafish	Kidney marrow (pronephros) Thymus	Primitive	VLM, RBI	Primitive macrophages and granulocytes (Herbomel et al., 1999)
			PLM, ICM	Primitive erythrocytes (Detrich et al., 1995; Thompson et al., 1998)
		Definitive (transient)	PBI	Erythromyeloid progenitors (mammalian EMP-like) (Bertrand et al., 2007)
				Lympho-myeloid and myeloid progenitors* (He et al., 2020) CD4 Tαβ lymphocytes (HSC-independent) (Tian et al., 2017)
		Definitive	VDA	
				HSC (Bertrand et al., 2010; Kissa and Herbomel, 2010)
Axolotl	Spleen Thymus	?	?	HSC? (Lopez et al., 2014)
Xenopus	Liver periphery (HSC) Spleen Bone marrow (GMP and lymphocytes) Thymus	Primitive Definitive Definitive (HSC)	aVBI pVBI DLP	Primitive erythrocytes (Ciau-Uitz et al., 2010) Definitive erythrocytes (Ciau-Uitz et al., 2010) HSC (Ciau-Uitz et al., 2010)
Chicken	Bone marrow Thymus Bursa of Fabricius	Primitive	YS	Macrophages and erythrocytes
		Definitive	Dorsal aorta	HSC (Yvernogeau and Robin, 2017)

BM, bone marrow; ICM, intermediate cell mass; HSCs, hematopoietic stem cells; PBI, posterior blood island; PLM, posterior-lateral mesoderm; VDA, ventral dorsal aorta; VLM, ventrolateral mesoderm; YS, yolk sac; VBI, ventral blood islands; DLP, dorsal lateral plate; GMP, granulocyte-macrophage progenitor. *Erythroid potential was not assessed for these progenitors.

The DA resembles the mammalian AGM and was first suggested to harbor hematopoietic stem cell activity based on the identification of transcripts for the zebrafish orthologs of *cmyb*, *lmo2* (Thompson et al., 1998), and *runx1* (Kalev-Zylinska et al., 2002). Co-expression of these markers with vascular markers (*fli1*, *flk1*, and *flk4*) suggested they are hemangioblasts (Thompson et al., 1998). Challenging this idea, the emergence of definitive HSCs in the DA was directly visualized as an EHT mechanism where cells in endothelial position and morphology adopted a spherical shape and migrated ventrally toward the caudal vein (Bertrand et al., 2010). This process did not require cell division and EHT was taken as direct evidence for HSC originating from HE (Kissa and Herbomel, 2010).

The endothelial lineage branch to create HE before cells migrate across the ventral somites to reach the ventral aspect of the dorsal aorta (Kobayashi et al., 2014). This process involves the expression of adhesion molecules (Jam) that ensure the required strength of Notch signaling delivered by the somite cells (Kobayashi et al., 2014). Attempts to dissect the molecular events that shape EHT identified Runx1 as an essential player inducing the survival of newly generated hematopoietic cells (Kissa and Herbomel, 2010). Bmp4 is expressed in the ventral aspect of the dorsal aorta and promotes the hematopoietic stem cell program, whereas the expression of Shh in the roof maintains the arterial program (Wilkinson et al., 2009). More recently, it was shown that myeloid cells (Espín-Palazón et al., 2014) and metabolic alterations that promote inflammasome-induced IL1β-signaling in macrophages enhance HSC production, and inflammasome inhibition results in decreased hematopoietic generation (Frame et al., 2020). Taken together, these observations indicate that the EHT is a complex process involving several key molecular players and different cell types. The sequence of events and the precise molecular requirements essential for EHT remain largely incomplete, and attempts to recreate the conditions to promote EHT *in vitro* are still being developed (Gomes et al., 2018).

**FIGURE 3:**
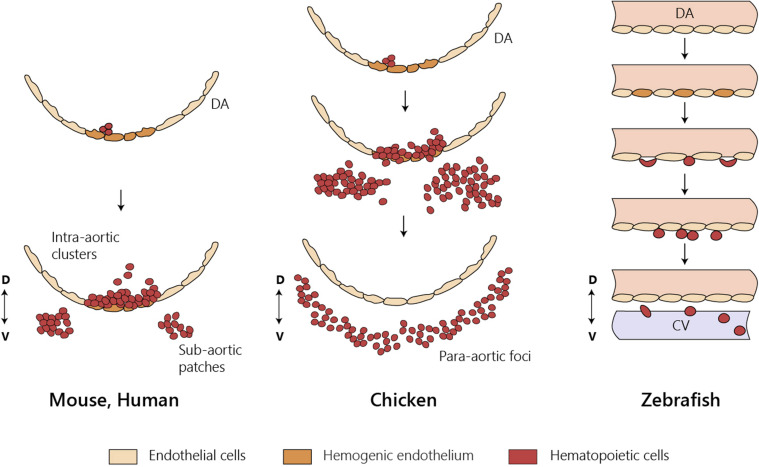
HSC emergence at the dorsal aorta in mice, chicken, and zebrafish. Intra-embryonic hematopoietic clusters (IAHCs) containing newly formed HSCs in birds and in mammals. IAHC originate from PAF in chicken and sub-aortic patches in mice. Contrary to mammals and birds, IAHCs are not observed in zebrafish and newly formed HSCs do not enter directly into circulation. CV, caudal vein; D, dorsal; DA, dorsal aorta; V, ventral; PAF, para-aortic foci.

Contrary to mammals and birds, intra-aortic clusters were not observed in zebrafish and newly formed HSCs did not directly enter circulation (Figure 3). HSCs migrate and reside transiently in the posterior region in the tail, called caudal hematopoietic tissue (CHT) (Murayama et al., 2006). At earlier stages, before 36 hpf, this region, which also generates hematopoietic progenitor cells, independently of HSCs (discussed below), is more commonly referred as posterior blood island (PBI) and corresponds to the ventral portion of the tail immediately caudal to the yolk tube extension (Bertrand et al., 2007). Like the mammalian FL, the CHT environment promotes the expansion and differentiation of newly formed HSCs. This niche is modulated by the incoming hematopoietic cells that remodel the vascular niche (Tamplin et al., 2015). Migration of the hematopoietic cells toward the CHT depends on CxCL8/CXCR1 chemokine signaling that also promotes residency (Blaser et al., 2017); Klf6a promotes their maintenance and expansion in the CHT through CCL25b-Ccr7 chemokine signaling (Xu et al., 2015).

Fate-mapping studies revealed erythromyeloid progenitors (EMP), analogous to the mammalian EMP, arise at 24–30 hpf in the vascular plexus of the PBI (Bertrand et al., 2007). These cells have both erythroid and myeloid differentiation potentials but lack lymphoid potential. In mice, it is currently accepted that the microglia (brain-resident macrophages) are exclusively derived from the YS (Ginhoux et al., 2010; Gomez Perdiguero et al., 2015). By contrast, in zebrafish, embryonic/larval and adult microglia were shown to have different sources: the RBI, the YS equivalent for the former and the ventral wall of the DA for the latter (Xu et al., 2015). These differences may be species-specific and/or reflect different timing in the establishment of the blood–brain barrier.

Fate-mapping analysis using high temporal–spatial resolution cell labeling techniques identified two waves of T lymphopoiesis. One, which is HSC-independent, originates in the DA and PBI regions and generates predominantly CD4 αβ T cells found only in the larval stage. A later HSC-dependent wave develops from progenitors restricted to the DA and gives rise to various types of T cells from the larval stage up to adulthood (Tian et al., 2017). Lymphopoiesis is initiated in the thymus after 3 days post-fertilization (dpf), with the onset of *rag1* expression, although the first progenitor immigrants arrive in the thymus by 54 hpf (Kissa et al., 2008). Live imaging of these progenitors revealed that besides extravasation from the nearest vessels, most of them migrate to the thymus through the mesenchyme with remote sites of extravasation (Kissa et al., 2008). By 4 dpf, hematopoietic cells seed the kidney marrow (pronephros), the BM counterpart in the zebrafish, where they establish the adult hematopoietic system (Murayama et al., 2006).

### Amphibians (Axolotl and *Xenopus*)

Two major amphibian models have contributed to our understanding of biological processes. One is the urodele *Axolotl mexicanum*, a model of tissue regeneration without scar tissue formation. The other is the anuran *Xenopus laevis* (allotetraploid) and *tropicalis* (diploid) that are the most-studied amphibian model systems for vertebrate embryonic development, cell and molecular biology, immunology, and, more recently, evolutionary diversification following genome duplication.

### Axolotl

The *Axolotl mexicanum* has been used to study tissue regeneration without fibrosis. Although little is known of its hematopoietic system, this is nevertheless an interesting model to study hematopoiesis because, unlike *Xenopus*, adult animals retain many embryonic features (neoteny) and do not undergo a clear stage of metamorphosis. The main hematopoietic organs were recently identified (Lopez et al., 2014). Using cell transplantation assays with minimal graft-vs.-host reaction, it was shown that the liver is the first site of hematopoiesis. High expression of the lymphocyte-specific enzyme terminal deoxynucleotidyl transferase (TdT) was found transiently in the liver and later being restricted to the spleen (Golub et al., 2004) where it remains throughout life. So, like in other model systems lacking hematopoietic BM, the spleen is the adult hematopoietic organ. These recent advances, however, did not indicate the origin of the hematopoietic cells that colonize these organs.

### Frogs

The *Xenopus* model has been extensively used to study hematopoiesis because larval chimeras are easy to make and early embryos have large cells that can be marked and traced in fate-mapping studies (Ciau-Uitz and Patient, 2016). Natural polyploids have also been used as models to study the regulation of gene duplication and gene silencing and to identify donor and recipient cells in transplantation experiments (Du Pasquier et al., 1989). Transplantation and chimera experiments indicated that hematopoietic cells originate first in the ventral blood islands (VBI) and later in the tissues originated from the dorsal lateral plate (DLP) (Chen and Turpen, 1995). Lineage tracing experiments established that these two territories are independent. Thus, blastomeres from 32-cell stage embryos that contribute to the DLP and to adult HSCs do not contribute to VBI, and conversely, blastomeres that contribute to VBI do not participate in the generation of adult HSCs (Ciau-Uitz et al., 2000). The VBI, the equivalent site to the mammalian YS, comprises an initial anterior region (aVBI) where primitive erythrocytes are first found along with myeloid cells and, at later stages, a posterior VBI (pVBI) that contains definitive erythrocytes, myeloid cells, and, similar to what was found in the zebrafish, also lymphocytes (Smith et al., 1989; Turpen et al., 1997). A third wave of hematopoietic generation occurs in the AGM, formed by the midline migration of the DLP, with the emergence of HSC and hematopoietic intra-aortic clusters (HIAC) formation.

It has been proposed that in aVBI, hemangioblast-like cells are at the origin of the first hematopoietic cells (Ciau-Uitz et al., 2010). By contrast, hemangioblast-like cells were not found in the pVBI, and hematopoietic cells are produced through an EHT process similar to that operating in fish, birds, and mammals in the DA and large arteries. Thus, hematopoiesis in frogs appears to occur in three independent waves similar to bony fish, birds, and mammals. Recently, a fourth myeloid cell generation has been described to occur in the mesenchyme posterior to the DLP (Imai et al., 2017).

The peripheral region of the liver was shown to be the primary hematopoietic organ throughout life containing HSC and erythroid progenitors, whereas the BM contains myeloid progenitors that respond to myeloid growth factors (Yaparla et al., 2019). Lymphocytes are produced in the thymus and the spleen in juvenile frogs, whereas the BM and the thymus have lymphopoietic activity in the adult (Greenhalgh et al., 1993).

### Birds

The large size and the easy manipulation of the avian fertilized egg allowed the construction of xenogeneic and congenic chimeras. The quail–chicken chimera system devised by Nicole Le Douarin combined with a rigorous identification of tissues originated from each species provided a reliable lineage-tracing tool (Le Douarin, 1969). Seminal experiments using this chimeric system established the intra-embryonic origin of HSC at a time when the YS was the consensual source of definitive hematopoiesis (Dieterlen-Lievre, 1975). Similar experiments using congenic chicken strains reinforced this notion (Lassila et al., 1978). The DA was soon designated as the site of origin of the hematopoietic progenitors of intra-embryonic origin because it harbored intra-aortic hematopoietic clusters (IAHC) comprising hematopoietic cells (Le Douarin and Dieterlen-Lièvre, 2013). IAHCs are the origin of another hematopoietic cell structure called para-aortic foci (PAF) formed later below the endothelial cell layer (Figure 3). A similar structure was described in the mouse and designated sub-aortic patches (Manaia et al., 2000). Cells within the IAHC or PAF contain HSC able of long-term reconstitution of the hematopoietic system (Dunon et al., 1998). Although it is presently difficult to phenotypically distinguish cells from the two structures, it is thought that PAF are an intermediate site where HSC mature before colonizing the thymus, the bursa of Fabricius, and the BM (Yvernogeau and Robin, 2017). The quail–chicken chimera system was also used to demonstrate the YS origin of most macrophages found in the central nervous system (microglia) (Cuadros et al., 1993).

Another important contribution from the Dieterlen-Lièvre group was the observation that two independent sources of endothelial cells contributed to the formation of the DA (Pardanaud et al., 1996). Thus, the roof and the sides of the DA are formed by endothelial cells originated in the somites whereas the floor of the DA, where hematopoietic cell generation occurs, is of splanchnopleural origin (ventral mesoderm). After hematopoietic cell generation, the endothelial cells in the floor of the DA are replaced by somite-derived cells similar to the remaining vessel wall (Pouget et al., 2006). This replacement process might not be evolutionarily conserved across vertebrates and is currently under investigation in other species. Additional experiments showed that expression of *Runx1* and activation of the Notch signaling pathway in HE required contact with the underlying mesenchymal cells. Both signals are essential for EHT, reinforcing the notion that EHT is an exceptional phenomenon that occurs under unique developmental conditions (Richard et al., 2013). Cell-labeling experiments targeting endothelial cells in embryos prior to IAHC emergence provided further evidence for an endothelial origin of the emerging hematopoietic cells (Jaffredo et al., 2000).

In summary, the chicken hematopoietic development also appears to occur in layers, although the precise contribution of YS to hematopoiesis has not been determined and no EMPs have been identified, either because they do not exist or because their identification was hampered by the absence of antibodies specific for the different hematopoietic lineages.

### Mice

The first hematopoietic cells arise in the mouse YS around E6.5–E7 in blood islands (Moore and Metcalf, 1970). Colony-forming assays identified bipotent erythroid/megakaryocyte (Xu et al., 2001; Tober et al., 2007) and macrophage progenitors (Palis et al., 1999) within these structures. Primitive erythroid progenitors (Ery-P) are exclusively present in the YS from E7.5 to E8.5 (Palis et al., 1999) and generate primitive erythrocytes. These primitive erythrocytes are larger than their BM counterparts and lack adult globin expression (Wong et al., 1986). Similar to other non-mammalian species, mouse Ery-P maintain their nucleus for several days (Kingsley et al., 2004). In contrast to those found in adult BM, primitive macrophages do not differentiate through a monocyte stage but directly from monopotent macrophage precursors (Mac-CFC/YS-Mp) (Bertrand et al., 2005).

The second hematopoietic wave starts at E8.5, with EMPs emerging from HE in the recently formed YS vascular bed, through a process of EHT (Goldie et al., 2008; Bertrand et al., 2010; McGrath et al., 2015; Frame et al., 2016; Kasaai et al., 2017). These progenitors proliferate extensively during the following 48 h and are found in circulation by E9 and in the hepatic primordium by E10.5 (Palis et al., 1999). This second wave generates definitive erythroid progenitors, the first mast cells, and bipotent macrophage granulocyte progenitors. Embryonic definitive erythrocytes can be found in circulation around E11.5–E12.5 and are distinguished from primitive erythrocytes by their small size (similar to adult erythrocytes) and the absence of nucleus. They originate from a novel population of progenitors of YS-EMP origin, recently identified, that ensures erythrocyte production and oxygenation up until birth (Soares-da-Silva et al. bioRxiv 2020.02.27.968230; doi: https://doi.org/10.1101/2020.02.27.968230).

Of note, the second hematopoietic wave generates tissue-resident macrophages (Gomez Perdiguero et al., 2015; Hoeffel et al., 2015). They have self-renewal ability and persist throughout life (Merad et al., 2002; Ajami et al., 2007; Jenkins et al., 2011).

The third and last wave of hematopoietic cell generation starts around E9.5, when multipotent progenitors emerge through EHT in the embryo DA and other large blood vessels (de Bruijn et al., 2000; Zovein et al., 2008; Boisset et al., 2010; Yzaguirre and Speck, 2016). Opposite to the first two hematopoietic waves, this third wave generates the progenitors of HSC and is the only capable to give rise to lymphocytes (Cumano et al., 1996; Medvinsky and Dzierzak, 1996). Similar to zebrafish, Notch signaling in the mouse AGM is determinant for hematopoietic cell generation (Kumano et al., 2003), and YS-derived macrophages, which are the most represented mature cells in this location, have been shown to enhance HSC emergence (Mariani et al., 2019). The total number of hematopoietic cells generated in the AGM has been estimated to be in the order of several hundreds of cells (Godin et al., 1999; Ganuza et al., 2017). According to some authors, HSC can also be generated in extraembryonic structures such as the placenta (Gekas et al., 2005; Ottersbach and Dzierzak, 2005). HSC then migrate to the FL where they mature, proliferate [expanding by more than 30-fold (Ema and Nakauchi, 2000)], and differentiate. Although adult and embryonic HSCs produce all major blood cell types, some specific lymphoid lineages are only produced during embryonic development, namely, the dendritic epidermal T cells (DETC) (Vγ5^+^) (Ikuta et al., 1990), lymphoid tissue inducer (LTi) cells (Eberl et al., 2004), and a subset of IL-17-producer γδ T cells (Vγ6^+^) (Haas et al., 2012).

### Humans

The development of the human hematopoietic system also follows a layered strategy. The first hematopoietic cells are found in the YS [starting at around E16–18, Carnegie stage (CS) 7–8]. At this stage, CD45^+^ cells are found in the cardiac cavities and mark the onset of circulation (Tavian et al., 1999). Whereas in mice the generation of hematopoietic cells and their detection in the FL and placenta are largely overlapping, in humans, the spatial–temporal progression of hematopoiesis is better discriminated (Ivanovs et al., 2017). In the YS, few megakaryocytes and macrophages are found in addition to erythrocytes. Although a population of EMP has not been formally identified, data suggests a second wave of hematopoietic cell emergence in the YS at around CS 13–15. IAHC emerge in the DA between CS 13 and 17 and CD34^+^CD45^+^ progenitors colonize the FL by CS 13, suggesting that HSC initiate liver colonization as they are generated (Tavian et al., 1996; Ivanovs et al., 2011). CD34^+^CD45^*lo*^ HSC are found in the placenta only after the 9th week of gestation (4 weeks after the first CD34^+^ cells reach this site). For this reason, the placenta is not considered a site of HSC generation in humans. The BM formation in humans marks the end of the embryonic period (CS 23 or E56) and colonization by HSC is initiated shortly after, although the exact time of HSC activity in the BM remains undetermined. Like in the mouse, human HSC initially express endothelial and hematopoietic markers that together with the detection of IAHC suggest a generation through an EHT. Curiously, and unlike in mice, the umbilical artery does not appear to be a site of hematopoietic cell generation in humans (Ivanovs et al., 2011).

### An Evolutionarily Conserved Design

From the comparison of hematopoietic development across species, it appears that the sequential generation of different hematopoietic progenitors was an evolutionarily successful strategy. Establishment of circulation was the key step toward the establishment of a vertebrate hematopoietic system.

Paradoxically, the first hematopoietic cells to be produced are differentiated rather than multipotent progenitors and fulfill the most basic functions like oxygen delivery, hemostasis, and tissue remodeling. Overall, despite some variations, distinct YS and DA hematopoietic generations are well conserved. Although controversial (Ueno and Weissman, 2006), the notion that bipotent hemangioblast-like cells contribute to hematopoietic cell generation is associated with that of primitive erythrocytes, megakaryocytes, and macrophages that occur in the YS blood islands.

With tissue complexity, subsequent hematopoietic waves have to fulfill increasingly specialized functions, and for that, multipotent progenitors are better adapted to generate a diverse array of cells with specific and diversified functions.

Remarkably, gene expression analysis in cephalochordates led to the identification of HE (Pascual-Anaya et al., 2013), which places EHT process as ancient and highly conserved through phylogeny. EHT appears to be the mechanism operating in the generation of multipotent progenitors in late YS and in the AGM and the only one to contribute to HSC production.

There is a consistent contribution of YS to the microglia and other tissue-resident macrophages and to primitive erythrocytes (Godin and Cumano, 2002). It is also generally accepted that YS hematopoietic cells do not contribute to the lymphoid compartment, although some minor and transient lymphocyte subsets have been suggested to emerge in the YS (discussed below). Other sites of hematopoietic cell generation, the most discussed being the placenta (Gekas et al., 2005; Ottersbach and Dzierzak, 2005; Rhodes et al., 2008), have been proposed but are not consensual, and the allantois that was hypothesized to be an alternative hematopoietic site in the chicken was recently shown to have no contribution to HSC generation (Yvernogeau and Robin, 2017). Recent evidence points to a role of YS-derived macrophages in the generation of HSC (Mariani et al., 2019). It is tempting to speculate that a selective pressure for an early generation of this unique type of macrophages lies not only in their capacity to ensure general tissue homeostasis, as the embryo develops, but also in their contribution to HSC emergence. It remains to be determined whether the unique properties of YS-derived macrophages are cell autonomous or whether they are modulated by cues received from the environment where they reside.

As different hematopoietic cells emerge, they transit through different primary hematopoietic organs: the FL and later the BM or their equivalents in the different species. Due to the pronounced differences in cell composition of the primary hematopoietic organs, it is to be expected that the cues that hematopoietic progenitors receive are substantially different in distinct organs and that this may influence their development. A recent example of this selective pressure is the lower requirements for erythropoietin (Epo) of embryonic erythrocytes compared to those in BM. They develop in the FL where Epo concentrations are lower than those produced in the kidney of adults. The recent discovery that these embryonic erythrocytes are of YS origin and outcompete those of HSC origin in the FL exemplifies how the environment can select for progenitors of different origins in space and time (Soares-da-Silva et al. bioRxiv 2020.02.27.968230; doi: https://doi.org/10.1101/2020.02.27.968230). The generally low concentration of other cytokines (i.e., IL-7) in FL also impacts lymphocyte development (see below).

## Lymphocyte Development: A Strategy Designed for Life-Long Tissue Homeostasis and Immune Protection

Lymphopoiesis, the process of lymphocyte generation, is the best-documented system where successive waves of progenitors differentiate and migrate to specific tissues thus generating the basis of a layered developmental organization. In this section, we will describe the development of embryonic lymphoid lineage populations and why they are selectively produced within an embryonic environment resulting in a diverse adult lymphoid compartment.

### B Cell Development

**FIGURE 4:**
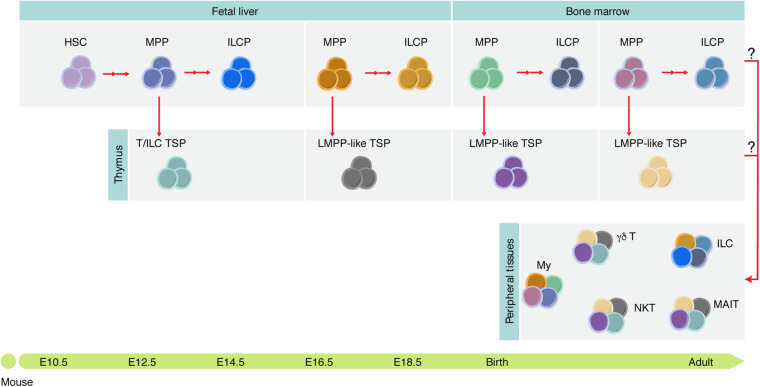
Tissue-resident immune cells are mosaic of cells derived at different developmental stages. Different hematopoietic progenitors are generated during development, some of which contribute to the adult tissue-resident immune cell compartment diversity. It is still unknown which functions these cells play during development and whether they persist in adulthood. ILCP, ILC progenitor; My, myeloid cells; NKT, NK T cells; MAIT, mucosal-associated invariant T cells.

The “layered immune system” concept was initially proposed by Herzenberg (1989), with the identification of functionally distinct “lineages” of B cells that are generated during ontogeny. B-1a cells, which reside mainly in the peritoneal and pleural cavities and produce natural antibodies in a T cell-independent manner, are usually obtained from embryonic precursors and contribute to innate-like immunity (reviewed in Herzenberg, 2000; Montecino-Rodriguez and Dorshkind, 2012; Elsaid et al., 2019). There are different explanations for the unique capacity of fetal progenitors to generate specific lymphoid cells (Figure 4).

The identification of a specific B1 progenitor only found in the embryo led to the hypothesis that these cells could derive from progenitors distinct from those that generate classical B-2 B cells. Recent studies show that innate-like B and T cells preferentially originate from developmentally restricted progenitors (Beaudin et al., 2016). These cells are exclusively found in fetal and neonatal stages, suggesting that they represent a distinct lineage from HSC.

However, experiments of cellular barcoding coupled with transplantation showed that FL HSCs can generate both B-1a and B-2 cells and that the capacity to generate B-1a cells is lost during development and upon transplantation (Kristiansen et al., 2016). Moreover, it was recently shown that antigen receptor specificity is sufficient to induce cell proliferation and acquisition of the B-1 phenotype and function (Graf et al., 2019). Lin28b, a regulator of fetal hematopoiesis, is important in the generation of B1a lineage, and loss of its expression controls the switch from neonatal to adult B cell production (Vanhee et al., 2019). Low levels of IL-7, a cytokine crucial for adult B cell development, in FL favors the generation of B1, highlighting the impact of the microenvironment on the preferential production of particular cell types (Wong et al., 2019).

### Thymopoiesis

In the thymus, T lymphocyte differentiation occurs from hematopoietic progenitors migrating from either the FL or the BM at later developmental stages in a process called “thymopoiesis.” In general, thymopoiesis resembles B lymphopoiesis in many ways, as it is also a stepwise process of rearrangement of antigen receptor genes and selection to ensure self-tolerance (Abramson and Anderson, 2017; Hosokawa and Rothenberg, 2020). The thymus produces T lymphocytes that are important for protection against environmental pathogens. Thymus architecture is key to T cell tolerance and a normal immune system. The first thymic seeding progenitors (TSP) that colonize the embryonic thymus display a unique capacity to generate innate and innate-like lymphocytes. These cells are required to shape the thymic architecture, thus ensuring life-long efficient T cell tolerance and avoiding autoimmunity (Elsaid et al., 2020).

The layered organization of thymopoiesis has been reported in all species studied from zebrafish to humans. During development, multiple waves of hematopoietic progenitors derived from distinct anatomical sites colonize the thymus. These waves give rise to T cells that fulfill distinct functions at distinct developmental stages. We will discuss below embryonic thymopoiesis across species and how cells from different waves contribute to diverse T cell compartments.

### Zebrafish

Fate-mapping analysis using high temporal–spatial resolution cell labeling techniques identified two waves of T cell progenitors: an early HSC-independent, from the DA and PBI regions, generating predominantly αβ T cells that do not persist beyond the larva stage and a late HSC-dependent T lymphopoiesis, restricted to the DA, giving rise to various types of T cells from the larval stage up to adulthood (Tian et al., 2017). This first wave shares two properties with the corresponding population in mice: they are important in thymic architecture and they differentiate fast (Hess and Boehm, 2012). In-depth characterization of the phenotype and function of T cells generated from this HSC-independent precursor is presently not available.

### Frogs

*Xenopus laevis* have two major hematopoietic organs, the liver, which is predominant in the larvae, and the spleen, the major hematopoietic organ in adult animals. B cells are produced in the liver in larval stages and the spleen takes over that function after metamorphosis (Du Pasquier et al., 1989). The diversity of the B cell repertoire is apparent in larvae and it is achieved through the combinatorial rearrangement of a large number VH, DH, and JH segments. Somatic hypermutation also operates in *Xenopus* larvae possibly as a response to the complex biota of their environment. Interestingly, terminal deoxynucleotidyl transferase (TdT) expression and N sequence additions are only detected after metamorphosis (Lee and Hsu, 1994), indicating further diversification of the antigen receptor repertoire (see Table 1) in adult animals.

Two waves of TSP, predominantly of DLP origin, have been identified during the larval stages. The first wave generates a transient population of T lymphocytes, whereas the progeny of the second wave persists in the adult (Turpen and Smith, 1989). It has been reported that T and B cells can also originate in the VBI (Smith et al., 1989). A third wave of progenitors enters the thymus at the beginning of metamorphosis and provides T cells throughout adult life.

In conclusion, T lymphopoiesis in frogs is organized in waves, and B and T lymphocytes with a limited repertoire diversity are produced early in embryonic life. The exposure of frog and fish larvae to a biota-rich environment compared to chicken and mammals might account for some differences in timing and the type of lymphocyte responses in the different species.

### Birds

An interesting and unique property of the chicken lymphoid development is that B cells develop in a unique environment called bursa of Fabricius where they acquire a diverse antigen receptor repertoire (Reynaud et al., 1987). Sequential transplantation of thymic lobes demonstrated for the first time that the embryonic thymus is discontinuously seeded by hematopoietic cells. In total, three waves of hematopoietic cells reach the thymus: the first at E6.5 and the third at E18, 1 day before hatching (Coltey et al., 1987). It was subsequently shown that the first wave of TSP is derived from cells located in the para-aortic clusters shortly after their generation in the DA. These observations raised the possibility that, in chickens, the first wave of TSP is composed of multipotent hematopoietic cells. The refractory periods where no hematopoietic progenitors reach the thymus are consequent to the absence of circulating progenitors because the injection in circulation of cells derived from para-aortic foci during refractory periods resulted in efficient thymic colonization (Dunon et al., 1998, 1999).

### Mice

**FIGURE 5:**
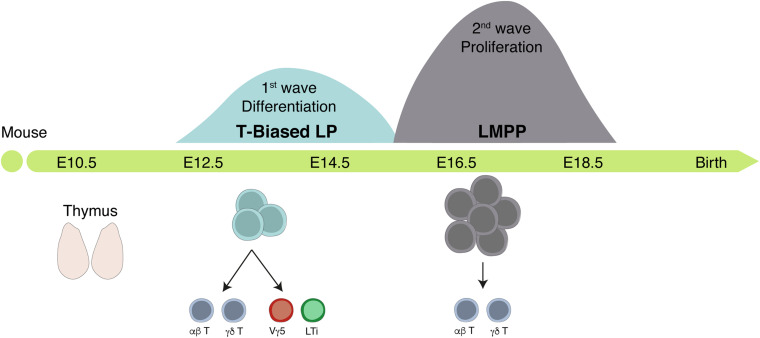
The fetal thymus is colonized by two successive waves of hematopoietic progenitors. Scheme of the timeline of thymus colonization in the mouse. Area of the Gaussian curves is proportional to the number of progenitors in each wave. The first wave generates few cells whereas the second expands before differentiation but lacks the capacity to generate LTi and Vγ5^+^ T cells.

Two waves of distinct TSP colonize the fetal thymus where they contribute to thymic organogenesis. First wave TSP are first detected around E12.5 and persist until E15.5. These progenitors differentiate rapidly, exhibit low proliferative capacity, express transcripts related to the ILC lineage, and are the only ones with the capacity to generate Vγ5^+^ T cells and LTi cells. A second wave of more immature hematopoietic progenitors colonize the thymus after E15 and ensure thymopoiesis thereafter (Figure 5; Ramond et al., 2014; Elsaid et al., 2020).

The thymic rudiment is detected around E11.5 in the mouse embryo (Takahama et al., 2017). The thymic epithelial cells (TEC) divide into cortical TEC (cTEC) and medullary TEC (mTEC). Aire^+^ mTECs ensure the expression of tissue-specific peptides that mediate T cell negative selection (see Table 1). Vγ5^+^ T cells and LTi cells, a subset of group 3 innate lymphoid cells (ILC3), are required to establish a mature compartment (*Aire*-expressing) of mTEC (Roberts et al., 2012). In line with this work, Elsaid et al. (2020) showed that the first wave of TSP generates invariant γδ T cells and LTi within a thymic microenvironment and that temporal depletion of this wave resulted in loss of (Aire)-expressing mTEC at birth (Figure 4). The crosstalk between the progeny of the first TSP and immature mTEC ensures that mature Aire^+^ mTEC are in time and place to induce tolerance (see Table 1) in the nascent αβ T cell repertoire during the neonatal period.

The first wave of TSP originates from a particular subset of FL common lymphoid progenitor (CLP), no longer detected after E15, that preferentially arises in the poor IL-7 environment of the FL (Berthault et al., 2017).

Before HSC activity, lympho-myeloid-restricted progenitors (LMP) co-expressing lymphoid- and myeloid-associated genes were reported in YS (Böiers et al., 2013) and proposed to be the origin of the first wave of TSPs (Luis et al., 2016). These studies challenged the notion that the first TSP derived from HSC relied on the analysis of reporter and lineage tracing mouse lines. Corroborating these observations, single-cell transcriptional and epigenetic analysis of IAHC detected a population of lympho-myeloid-primed progenitors, suggesting an early differentiation process or an HSC-independent generation (Zhu et al., 2020). Alternatively, or complementarily, despite a transient expression of lymphoid transcripts, the reported YS-derived LMP were devoid of lymphoid potential and therefore do not qualify as lymphoid progenitors (Elsaid et al., 2020).

### Humans

Growing evidence suggests that, as in mice, the human fetal thymus is colonized by distinct waves of hematopoietic progenitors. Unlike their adult counterpart (Tieppo et al., 2020), fetal thymic progenitors expressing low levels of TdT generate invariant γδ T cells and promote tolerance (Mold et al., 2010; Ng et al., 2019). It was further shown that, like that of innate B-1 cells in mice, the generation of fetal invariant γδ T cell subsets was Lin28b-dependent. Further studies are required to identify the phenotype and functions of these fetal-derived T cells in fetal immunity and organ development.

### Why a Layered Lymphoid Compartment?

Similar to what was described for hematopoietic cells, not all lymphocyte subsets are produced simultaneously nor throughout life. In the mouse, whereas lymphocytes with a highly variable receptor repertoire are produced constantly after birth, some particular subsets that comprise B1 B cells and Vγ5^+^ and Vγ6^+^ T cells are exclusively or preferentially produced during embryonic life and persist in adults. These subsets share some general properties: (1) they express invariant or restricted antigen receptor repertoires and are, therefore, not devoted to highly specific immune responses. Rather, they fulfill broader functions and are, therefore, commonly designated as “innate-like” cells; (2) they are produced in small numbers; and (3) rather than circulating, they reside in tissues. These lymphocyte subsets that might confer a first line of protection before adaptive immune cells are fully functional also contribute to tissue architecture and homeostasis. Due to their stage-restricted development, it is tempting to hypothesize that these particular lymphoid subsets originate from specialized cells that develop independent from HSC. The demonstration that B1 and B2 cells share a similar cellular origin (Kristiansen et al., 2016), that Vγ5-expressing T cells do not differentiate from conventional YS progenitors, and that embryonic hematopoietic progenitors often express lymphoid specific transcripts, regardless of their differentiation potential, offer alternative possibilities that need to be further investigated (Elsaid et al., 2020).

B1 cells make natural circulating immunoglobulin that provides a first line of defense against pathogens. Although B2 cells can also produce IgM, their development is delayed and transient protection is better achieved through immunoglobulins of B1 cell origin. In the absence of Vγ5^+^ T cells in the skin, wound healing is delayed (Jameson et al., 2002). Similarly, in the absence of Vγ6^+^ T cells, there is increased susceptibility to airway viral infections as epithelium integrity is easily compromised (Guo et al., 2018). Because IL-17, the main effector cytokine in epithelium regeneration, can also be produced by conventional T cells, γδ T cells might only be crucial in neonates and juveniles before conventional T cell immunity is mature. Both Vγ5^+^ T cells and LTi that contribute to the maturation of mTEC in the thymus have a common origin in specialized TSP only present in the embryo. Other cell types such as CD4^+^ T cells can produce RANKL and induce mTEC maturation. LTi are no longer found in the adult thymus, suggesting that, together with γδ T cells, they fulfill a very specific role in late gestation and around birth. The fact that they originate from a particular progenitor cell arising specifically in the low-IL-7 FL environment (Berthault et al., 2017) and that in some mouse strains neonatal tolerance induction is required for prevention of autoimmune disease later in life (Guerau-de-Arellano et al., 2009) suggests a role of these developmental-restricted cells in the establishment of peripheral tolerance.

A selective pressure including environmental constraints appears to favor the early development of less diverse and sophisticated immune cells that are important in the establishment of basic immune functions and that confer protection before the highly complex diverse immune system is operational. An early development before large numbers of lymphoid cells circulate also ensures an efficient colonization of peripheral tissues where niches are possibly limited. Cell-autonomous mechanisms that condition the differentiation of developmentally restricted progenitors are still largely unknown. The HSC-independent origin proposed by some authors will have to be probed against a possible loss of the expression of embryo-specific genes such as loss of Lin28b and of the B1 differentiation capacity as HSC transit from FL to BM, through either an internal clock-type mechanism or environmentally induced.

## Concluding Remarks

It is interesting to realize that cells usually considered as pro-inflammatory such as macrophages have been also involved in organogenesis and in maintenance of tissue integrity, throughout evolution. It follows that what is considered to be the adult inflammasome might have tissue-remodeling functions during development and that therefore both functions co-evolved. This notion can be extended to specific subsets of innate-like T cells and ILCs.

The first waves of immune cells appear to be also devoted to tissue remodeling although they can also engage in defense, namely, in species whose embryos are released in microbiota-rich environment. However, they appear to be prone to mount low inflammatory responses compatible with the integrity of tissues, as elsewhere described in *X. laevis* tadpoles.

## Author Contributions

MP and FS also contributed to the figures. AB, AC, FS, and RE conceptualized and assembled the manuscript. All authors reviewed the literature, discussed, contributed to writing, and approved the submitted version.

## Conflict of Interest

The authors declare that the research was conducted in the absence of any commercial or financial relationships that could be construed as a potential conflict of interest.

## Abbreviations

a: anterior
AGM: aorta gonads mesonephros
BM: bone marrow
BMP4: bone morphogenic protein 4
c: cortical
CFC: colony-forming cells
CHT: caudal hematopoietic tissue
CLP: common lymphoid progenitor
CS: Carnegie stage
DA: dorsal aorta
DETC: dendritic epidermal T cells
DLP: dorsal lateral plate
dpf: days post-fertilization
E: embryonic day
EHT: endothelial-to-hematopoietic transition
EMP: erythromyeloid progenitors
Ery-P: primitive erythroid
Epo: erythropoietin
ES: embryonic stem
FL: fetal liver
HE: hemogenic endothelium
hpf: hours post-fertilization
HSC: hematopoietic stem cells
HIAC: hematopoietic intra-aortic clusters
ICM: intermediate cell mass
IL: interleukin
LMP: lympho-myeloid-restricted progenitors
LTi: lymphoid tissue inducer
m: medullary
p: posterior
PAF: para-aortic foci
PBI: posterior blood islands
PLM: posterior-lateral mesoderm
RA: retinoic acid
RBI: rostral blood islands
Shh: sonic hedgehog
TdT: terminal deoxynucleotidyl transferase
TEC: thymic epithelial cell
TSP: thymic seeding progenitors
VBI: ventral blood islands
YS: yolk sac.
